# Risks of postoperative paresis in motor eloquently and non-eloquently located brain metastases

**DOI:** 10.1186/1471-2407-14-21

**Published:** 2014-01-14

**Authors:** Thomas Obermueller, Michael Schaeffner, Julia Gerhardt, Bernhard Meyer, Florian Ringel, Sandro M Krieg

**Affiliations:** 1 Department of Neurosurgery, Technische Universität München, Ismaninger Str. 22, 81675 Munich, Germany

**Keywords:** Cerebral metastases, Intraoperative neurophysiological monitoring, Motor evoked potentials, Neurological deficit

## Abstract

**Background:**

When treating cerebral metastases all involved multidisciplinary oncological specialists have to cooperate closely to provide the best care for these patients. For the resection of brain metastasis several studies reported a considerable risk of new postoperative paresis. Pre- and perioperative chemotherapy (Ctx) or radiotherapy (Rtx) alter vasculature and adjacent fiber tracts on the one hand, and many patients already present with paresis prior to surgery on the other hand. As such factors were repeatedly considered risk factors for perioperative complications, we designed this study to also identify risk factors for brain metastases resection.

**Methods:**

Between 2006 and 2011, we resected 206 brain metastases consecutively, 56 in eloquent motor areas and 150 in non-eloquent ones. We evaluated the influences of preoperative paresis, previous Rtx or Ctx as well as recursive partitioning analysis (RPA) class on postoperative outcome.

**Results:**

In general, 8.7% of all patients postoperatively developed a new permanent paresis. In contrast to preoperative Ctx, previous Rtx as a single or combined treatment strategy was a significant risk factor for postoperative motor weakness. This risk was even increased in perirolandic and rolandic lesions. Our data show significantly increased risk of new deficits for patients assigned to RPA class 3. Even in non-eloquently located brain metastases the risk of new postoperative paresis has not to be underestimated. Despite the microsurgical approach, our cohort shows a high rate of unexpected residual tumors in postoperative MRI, which supports recent data on brain metastases’ infiltrative nature but might also be the result of our strict study protocol.

**Conclusions:**

Surgical resection is a safe treatment of brain metastases. However, preoperative Rtx and RPA score 3 have to be taken into account when surgical resection is considered.

## Background

Today, treatment of cerebral metastases is a topic, which concerns many specialties and an interdisciplinary oncological cooperation is crucial to provide the best care for these patients. Modern treatment options for cerebral metastases limit surgical treatment to a subgroup of patients, which present with symptomatic lesions such as rolandic or cerebellar metastases. Both radiosurgery and surgical resection have been shown to have comparable rates of local control. By contrast, whole brain radiation therapy (WBRT) alone without surgery or radiosurgery led to significantly shorter survival and local control [1,2]. Nonetheless, many patients with supratentorial metastases show a focal deficit due to focal mass effects. These patients are especially eligible for surgical resection to facilitate early recovery from neurological deficits [3]. Thus, surgical resection frequently treats metastases within or close to the motor cortex or corticospinal tract (CST).

Just recently, there are some hints that cerebral metastases infiltrate surrounding brain tissue, which might change the surgical and radiosurgical approach [4,5]. Moreover, the medical and surgical community must discuss postoperative impairment of the motor system to properly select patients for surgical resection and to increase awareness of postoperative motor deficits during metastasis resection especially when the CST is infiltrated [2,6,7].

This study aims to identify risk factors for patients with brain metastases undergoing surgical resection, to raise awareness of those factors and encourage the proper selection of patients for surgical treatment.

## Methods

### Patient cohort

Between 2006 and 2011 206 patients underwent resection of brain metastases. An interdisciplinary tumor board discussed every case prior to surgery, and surgery required the consent of all disciplines (neurooncology, neurosurgery, medical oncology, and radiation oncology) with regard to the present treatment guidelines [8,9]. This board frequently recommended surgical resection, especially for patients with disabling motor weakness, increased edema formation, cystic metastases, or metastases resistant to radio- or chemotherapy.

**Figure 1:**
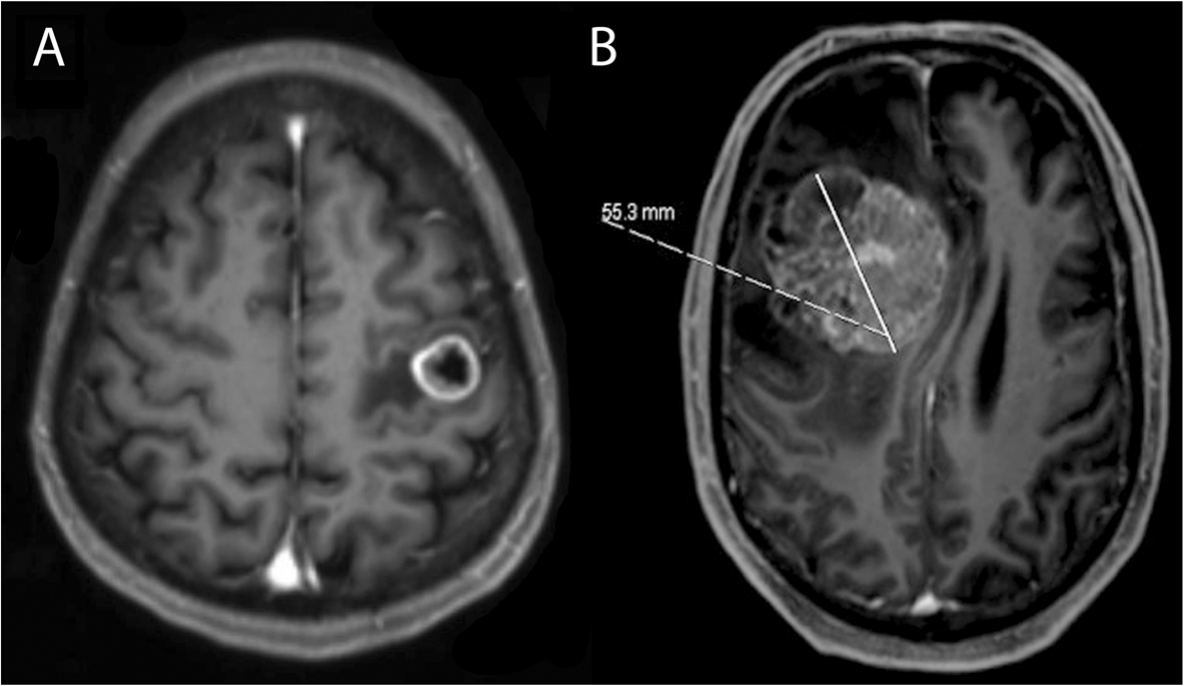
**Illustrative cases.** Examples of motor eloquently localized metastases in the precentral **(A)** and non-motor-eloquently localized metastases in the middle frontal lobe **(B)** as evaluated in this study. We also measure tumor diameter **(B)**.

These were 56 metastases in eloquent motor areas (in or directly adjacent to the rolandic cortex or CST) using intraoperative neurophysiological monitoring (IOM) by monopolar direct cortical stimulation for motor evoked potentials (MEPs) and 150 patients with metastases in non-eloquent brain regions in terms of motor function (all others), which underwent surgery without IOM. Figure 1 shows examples of the evaluated motor eloquent lesions. We determined eligibility for IOM based on the topographic association between metastases and CST or preoperative magnetic resonance imaging (MRI) of the primary motor cortex.

### Standardized patient evaluation

Prior to surgery, all patients underwent MRI for tumor diagnosis, localization, and acquisition of a navigational dataset for intraoperative neuronavigation (BrainLAB Vector Vision Sky, BrainLAB Vector Vision 2® or BrainLAB Curve®, Feldkirchen, Germany) (Figure 1). All patients underwent preoperative neurological evaluation of sensory function, muscle strength, coordination, and cranial nerve function. Each patient also received a recursive partitioning analysis (RPA) classification [10]. This score assigns patients with cerebral metastases to 3 classes:

Class 1: Karnofsky Performance Score (KPS) ≥70, age <65 years, controlled primary tumor, no extracranial metastases

Class 2: KPS ≥70 but not all of the above features

Class 3: KPS <70 [10].

All patients again underwent neurological assessment directly after anesthesia and daily from the first postoperative day until discharge. Routine follow-up included neurological assessment at 6–8 weeks postoperatively, and every 3 months on a regular basis.

Every patient who presented with a new paresis directly after surgery underwent an immediate cranial imaging to exclude secondary hemorrhage or ischemia, an MRI scan within 48 hours after resection to assess tumor removal, edema formation, and hemorrhage. Residual tumor was defined as any suspected contrast enhancement in the resection cavity on the MRI scan within 48 hours after surgery. The MRI protocol also included diffusion images to detect potential ischemia. Routine follow-up included MRI scans every 3 months, depending on concurrent oncological therapy and tumor entity. We also reviewed these follow-up MRI scans for recurrent metastases, since neurological status during follow-up was only considered during progression-free survival.

For further analysis, any new postoperative paresis was differentiated between permanent and temporary deficit. A new permanent paresis was defined as a new or aggravated motor deficit due to surgery that did not return to the preoperative status during follow-up. A temporary deficit was present when a new or aggravated postoperative paresis resolved at least during the regular 8-week follow-up interval. A motor deficit was defined as any impairment of motor performance even if the patient only presented with deteriorated fine motor skills of the small hand muscles.

### Surgical procedure

In general, we used total intravenous anesthesia (TIVA), and strictly avoided volatile anesthetics because of their interference with IOM [11-13]. Propofol and remifentanyl were continuously administered for intraoperative anesthesia and analgesia. We used the neuromuscular blocker rocuronium for intubation only and not during surgery.

As reported earlier, we used IOM by MEP monitoring in 56 cases, when tumor location was supposed to be close to the rolandic cortex or CST on axial slices of the preoperative MRI scans [3].

During resection, an amplitude decline of 50% or more of the baseline was considered a substantial decline. The surgeon reversed the causal surgical step when applicable, such as by removing spatulas, and irrigated the exposed brain with warm Ringer’s solution. When the tumor resection was close to major blood vessels, we irrigated the resection cavity with nimodipine to reverse or avoid vasospasm. In most cases, after stabilization or renormalization of MEPs, tumor resection proceeded [3].

### Ethical standard

The study is well in accordance with the ethical standards of the Technical University of Munich, the local ethics committee (registration number: 2826/10), and the Declaration of Helsinki.

### Statistical analysis

We performed a Chi-square test or Fisher’s exact test on the distribution of several attributes. Several tests yielded differences between two groups: the Mann–Whitney-Wilcoxon test, using multiple comparisons on ranks for independent samples, the Kruskall-Wallis test for nonparametric one-way analysis of variance (ANOVA), and Dunn’s test as the post hoc test. All results are presented as mean ± standard deviation (SD). We also calculated median and range (GraphPad Prism 5.0c, La Jolla, CA, USA); p < 0.05 was considered significant.

## Results

**Figure 2:**
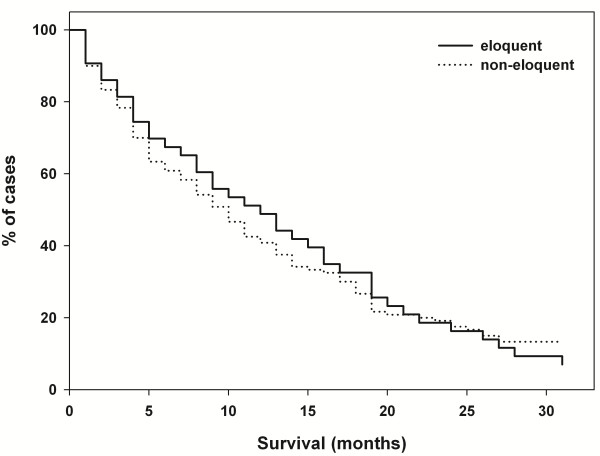
**Survival.** Kaplan-Meier survival analysis of motor eloquently and non-eloquently located brain metastases.

Out of 206 enrolled patients, 56 suffered from motor eloquent brain metastases and 150 from non-eloquently located lesions in terms of motor function. Details specific to preoperative status are shown in Table 1. The presence of motor eloquence had no statistically significant impact upon survival (Figure 2).

**Table 1:** Patient characteristics

		**Eloquent**	**Non-eloquent**
Number of patients		56	150
Preoperative paresis		57.0%	47.3%
Sex	Male	32 (57.0%)	72 (48.0%)
	Female	24 (43.0%)	78 (52.0%)
Median age ± SD		61.4 ± 13.1 years	60.9 ± 11.9 years
Location	Precentral	32.0%	-
	Frontal	27.0%	30.0%
	Subcortical	22.0%	-
	Postcentral	14.0%	-
	Cerebellar	-	20.0%
	Occipital	-	15.3%
	Temporomesial	-	13.3%
	Parietal w/o postcentral gyrus	-	9.3%
	Other	-	12.1%
Primary tumor	NSCLC	30.4%	29.5%
	Breast	21.4%	20.1%
	Melanoma	8.9%	10.7%
	Colon	7.1%	8.7%
	RCC	7.1%	10.1%
	CUP	7.1%	4.0%
	Ovarian	5.4%	2.7%
	Esophageal	3.6%	4.7%
	Seminoma	1.8%	0.7%
	Paranasial sinus	1.8%	0.7%
	Urothelial	1.8%	0.7%
	SCLC	1.8%	2.0%
	Uterine sarcoma	1.8%	0.7%
	Gastric	-	1.3%
	Larynx	-	1.3%
	Gall bladder	-	0.7%
	Parotid gland	-	0.7%
	Prostate	-	0.7%
Number of brain metastases	1	57.0%	56.7%
	2	18.0%	18.0%
	3	11.0%	10.3%
	>3	14.0%	15.0%
RPA score	1	14.0%	15.0%
	2	64.0%	65.0%
	3	22.0%	20.0%
Preoperative therapy	Rtx	5.4%	2.7%
(n = 150)	Ctx	30.4%	34.0%
	Rtx + Ctx	14.3%	19.3%

Overview of all enrolled patients including sex, preoperative existing deficit, primary tumor, and preoperative therapy). *Ctx*, Chemotherapy; *CUP*, Carcinoma of unknown primary; *NSCLC*, Non small cell lung cancer; *RCC*, Renal cell cancer; *Rtx*, radiotherapy; *SCLC*, Small cell lung cancer; *SD*, Standard deviation.

### Postoperative results

#### Motor eloquent tumor location

**Figure 3:**
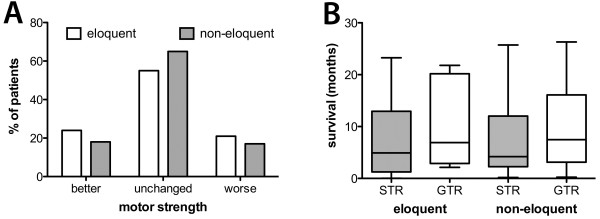
**Clinical course. A**: Columns showing the relation of motor eloquence of tumor and pre- and postoperative status. **B**: Correlation of survival in months with resection in postoperative MRI.

Out of the 56 patients with metastases in motor eloquent locations, 12 (21.4%) showed aggravated paresis after surgery, which remained permanent in seven (12.5%) and resolved during follow-up in 5 patients (8.9%) (Figure 3A). Among those seven with permanent deficits, four suffered from secondary hemorrhage, and three from ischemia. Moreover, postoperative MRIs on the five patients with temporary deficits revealed one case of ischemia and another two involving the supplementary motor area (SMA).

Thirteen patients (23.2%) improved after surgery. During the operation, the surgeon expected gross total resection (GTR) in 92.5% of cases and subtotal resection (STR) in 7.5%. However, postoperative MRI were searched for contrast enhancement and showed GTR in 72.0%, leaving an unexpected residual (UR) of 20.5%; at least according to the strict protocol of this study.

Cases with GTR had a mean survival of 10.6 months ± 8.9 months, in contrast to cases with STR 6.1 months ± 5.7 months (p = 0.31; Figure 3B). The mean survival after surgery was 8.3 ± 7.1 months (range 0.1-23.0 months).

#### Non-motor-eloquent tumor location

In general, 11 patients (7.0%) suffered from a permanent and six patients (4.3%) from temporary paresis in the non-motor-eloquent group. Among 11 patients with permanent deficits, one suffered from secondary hemorrhage and another from ischemia after surgery. Moreover, among the six with temporary increased paresis, one suffered from secondary hemorrhage and also underwent surgical revision.

Out of 150 patients, 27 (18.2%) showed improvement of their preoperative motor deficit (Figure 3A). During the operation, the surgeon expected GTR in 131 cases (89.3%) and STR in 15 cases (10.7%). We gathered postoperative MRI data in 117 cases, which showed GTR in 78 cases (66.7%) and STR in 39 cases (33.3%) according to our considerably strict study guidelines. Thus, an UR was present in 26 cases (22.6%). For the remaining manuscript, GTR is defined as MRI-confirmed GTR.

After GTR, overall survival was 9.1 ± 6.9 months and 7.5 ± 7.5 months after STR (p = 0.08). Concerning all patients harboring non-motor-eloquent metastases, the mean survival was 7.9 ± 8.2 months (range 0.5-47.0 months) after surgery (Figure 3B).

#### Histology and tumor location vs. new postoperative deficit

Type of primary tumor bore no significant relation to postoperative incidence of temporary or permanent impairment of motor function (data not shown). In the motor eloquent group, proximity to the rolandic cortex showed a trend to be associated with postoperative paresis (p = 0.101). Surgery of frontal cortex lesions anterior to the precentral gyrus never caused deficits, even in the insula, whereas resection within the precentral cortex had the strongest association with permanent deficits (23.5%).

**Figure 4:**
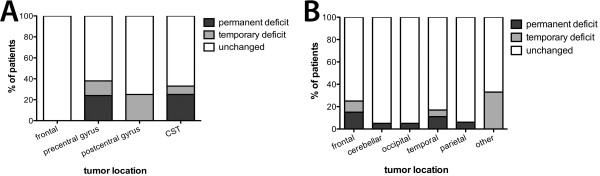
**Tumor location.** Columns represent the distribution of postoperative outcome in relation to metastasis location in motor eloquent **(A)** and non-eloquent **(B)** metastases. A trend towards postoperative deficits in eloquently located lesions is shown without reaching statistical significance (p = 0.101).

The non-motor-eloquently located group presented a wide variety of locations, and postoperative motor deficits were far less frequent in regions far away from the motor cortex or CST (p < 0.05; Figure 4). However, three patients showed permanent new paresis despite temporal tumor location. One of these patients suffered secondary hemorrhage, and one with temporodorsal metastasis presented with new ischemia within lateral parts of the CST.

#### RPA class

**Figure 5:**
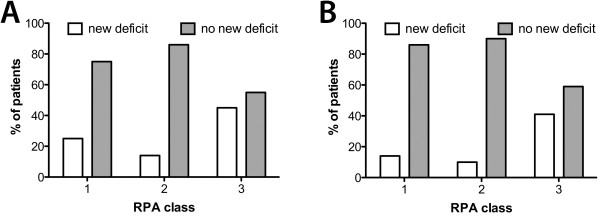
**Recursive partitioning analysis.** There was significant correlation between the RPA class and new postoperative deficit (eloquent **(A)**: p < 0.05; non-eloquent **(B)**: p < 0.001).

In the motor eloquent group, two patients (25.0%) of RPA class 1, five patients (13.9%) of class 2, and five patients (45.5%) of class 3 suffered new postoperative paresis (p < 0.05, Figure 5A). In patients with non-motor-eloquently located brain metastases, three patients (13.6%) of class 1, 10 patients (10.3%) of class 2, and 12 patients (41.4%) of class 3 showed a new postoperative paresis (p < 0.001; Figure 5B). However, there was no difference between motor eloquent and non-eloquent tumor location. Table 2 shows the results of the relation between RPA class and overall survival.

**Table 2:** Recursive partitioning analysis

**RPA-class**	**Eloquently located (months)**	**Non-eloquently located (months)**
**1**	9.9 ± 5.6	9,2 ± 5.9
**2**	9.1 ± 6.8	8.7 ± 7.7
**3**	4.8 ± 8.4	5.6 ± 9.7
**p-value**	0.411	0.279

Despite missing statistical significance (p = 0.41 and p = 0.28) a trend is shown towards a prolonged survival in RPA class 1 and 2 for all patients.

### Preoperative vs. postoperative deficit

**Figure 6:**
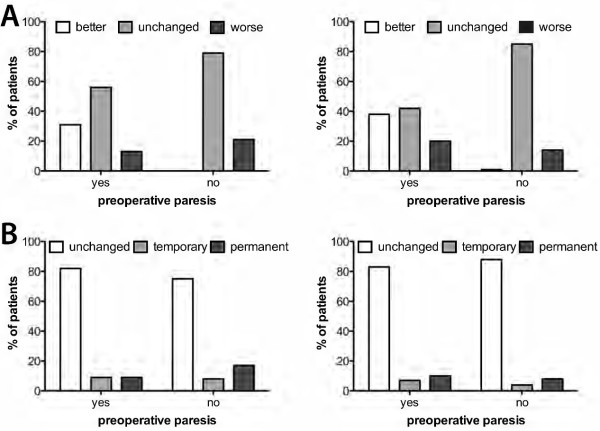
**Motor status. A**: Change in motor function after surgery in relation to the preoperative neurological status. **B**: Course of neurological status during follow-up. There was no significant relation between **A** and **B** in either group.

Regarding the rate of preoperative motor deficits and their effect on outcome, we found comparable results in both groups. Improvements emerged in 31.0% of the eloquent group and 38.0% in the non-eloquent group. Immediately after surgery, 13.0% of patients in the motor eloquent group with preoperative paresis deteriorated, compared to 21.0% in the motor eloquent group without preoperative paresis. In the non-eloquent group, we found deterioration in 20.0% with preoperative deficit and 14.0% without it (Figure 6A). Even in the follow-up, there was no significant difference between preoperative apparent and new postoperative deficit (Figure 6B).

### Preoperative therapy

**Figure 7:**
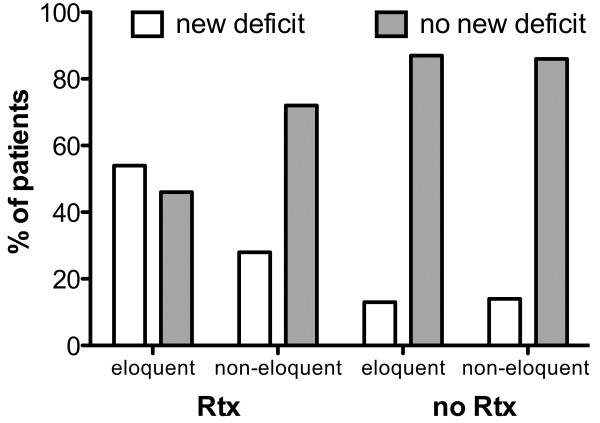
**Preoperative radiotherapy.** There is a significant difference in the occurrence of new postoperative deficits between patients treated by preoperative radiotherapy (Rtx) and patients who do not receive such treatment, in motor eloquent and non-eloquently located metastases.

Because some patients had received different treatments prior to surgery, we investigated the relation of preoperative treatment to postoperative deficit. In the motor eloquent group, 55% of patients who had had radiotherapy of the brain (Rtx) developed a new postoperative deficit, whereas patients who had had no Rtx developed it in only 13.0% of cases (p = 0.01; Figure 7A). In the non-motor-eloquent group, treatment with Rtx preceded a new deficit in 28.1% of cases, and no such treatment preceded it in 14.0% of cases (p < 0.05; Figure 7B). Preoperative chemotherapy (Ctx) had no significant effect on postoperative outcome. In motor eloquently located metastases, the occurrence of a new paresis was 24.0% with Ctx and 19.4% without it. In patients with non-motor-eloquently located metastases, 18.8% with and 14.7% without Ctx presented new pareses.

Table 3 represents the different preoperative strategies of all enrolled patients. Both groups showed statistically significant correlation between preoperative treatment and new postoperative paresis (motor eloquent: p = 0.012; non-eloquent p = 0.045).

**Table 3:** Preoperative therapy

		**Rtx**	**Ctx**	**Rtx + Ctx**	**No preoperative therapy**	**p**
**Eloquent**	New deficit	33.3%	5.9%	63.0%	17.8%	0.012
	No new deficit	66.6%	93.1%	37.0%	82.2%	
**Non-eloquent**	New deficit	0.0%	8.9%	33.3%	12.5%	0.045
	No new deficit	100%	91.1%	66.6%	87.5%	

All types of preoperative therapy had a significant effect on new postoperative deficits in the eloquent group (p = 0.012) and the non-eloquent group (p = 0.045). *Rtx*, Radiotherapy; *Ctx*, Chemotherapy.

## Discussion

### Outcome after brain metastasis surgery

Among all 206 patients, 39 (19.0%) improved their neurological status postoperatively, whereas 29 (14.0%) developed a new postoperative deficit. The number of deficits is comparable to that of other published studies which reported neurological deterioration in 6% (RPA score 1 and 2) and 19% (RPA score 3) of patients [4,14], even to stereotactic radiosurgical investigations [15,16]. We examined every patient meticulously and even mild deficits were taken account. The incidence of postoperative permanent deficit in the motor eloquently located group was higher than in the non-eloquent group (12.5% vs. 7.0%) due to the lesions’ adjacency to the motor cortex or subcortical motor tracts [3]. Surgery is comparable to radiosurgery in terms of local control and survival and still plays an indispensable role in the treatment of brain metastases [4,17,18].

### Analysis of postoperative MRI

#### Residual tumor

The residual measured in the postoperative MRI was about 20%, with a slight trend towards less residual in monitored patients. Use of IOM could explain this trend. Neurosurgeons have always considered a metastasis as a tumor with sharp borders. But recent studies provided some data that metastases instead might have an infiltrative growth pattern [4,19]. Our results of an unexpected residual of about 20% lead into the same direction, but we have to keep in mind that the definition of residual tumor presented by residual contrast enhancement can result in considerable overestimation of real UR due to reactive postoperative changes. We performed postoperative MRIs on 124 out of 150 patients in the non-eloquent group (82.6%), and 50 out of 56 patients in the motor eloquent group (89.0%). Lee et al. reported significant difference in survival between GTR (20.4 months) and STR (15.1 months)[18]. However, since that study included only patients initially treated by surgery, the results cannot be compared to our cohort.

As other studies have stated, tumor residual can lead to higher local recurrence rate and shorter survival. These studies improved the clinical outcome by performing supramarginal resections of metastases, even if eloquently located [4,5]. In the non-motor-eloquent group, a trend toward longer survival without tumor residual was shown (Figure 3B). In the motor eloquent group, that trend was even larger, but did not reach statistical significance (eloquent: p = 0.31; non-eloquent: p = 0.08). This might be due to different adjuvant strategies in individual patients and small sample size.

However, combining our results with the cited previous data, we have to consider whether intraoperative imaging or repeated resection have to be standard of care as both means should improve the rate of GTR and therefore survival.

#### Histology and tumor location

We found the type of primary tumor did not help predict new postoperative paresis or survival. Lagerwaard et al. described it as predictive of survival in their analysis of 1292 patients [20]. Hall et al.’s study of 740 patients also showed patients suffering ovarian cancer had the highest survival rate, small cell lung cancer patients the lowest [21]. Thus, it seems likely that our cohort is too small to show statistical significance in this matter.

However, our results confirm relation between proximity to the motor cortex and a considerably high risk of new postoperative motor deficit (Figure 4). Our number of postoperative deficits is high, partly because the standardized neurological evaluation of our patients defines even minor weakness as new paresis.

In the motor eloquent group, four patients suffered secondary hemorrhages causing permanent motor deficits. Ischemia only occurred in one case. When operating especially near or within the rolandic cortex, our department rarely uses the bipolar cautery, to avoid consecutive ischemia.

Even in the non-motor-eloquent group, we had two cases of new permanent motor deficits (1.3%) after surgery due to ischemia and secondary hemorrhage. This tells us that even such tumors carry the risk of postoperative paresis, and we have to bear this fact in mind when we counsel our patients.

#### RPA class vs. outcome

Patients in RPA class 3 had a significantly higher risk of new postoperative deficits (Figure 5). Besides the prognosis of survival, we can get information about the postoperative outcome. The most relevant factor in the RPA class system is the KPS. Patients were assigned to class 3 if they have a KPS below 70, regardless of other factors. In 2007, Eichler et al. recommended a KPS above 70 for surgical treatment [22].

The group of Schödel et al. investigated the impact of surgical resection on neurological outcome in 206 patients [23]. Poor RPA class was also detected as an independent indicator of shorter survival, but nevertheless, surgery can improve neurological status significantly in these cases, as our data clearly shows (Figure 3). Concerning patients after Rtx, Gaspar et al. investigated 1200 patients, and, in another study, 445 patients, and reported a significant difference in survival of 2.3 months in class 3, 4.2 months in class 2 and 7.1 months in class 1 [10,24]. Due to our small number of patients we failed to show statistical significance, but we did show a longer survival rate in surgically treated patients, as well as a trend towards longer survival in lower RPA classes (Table 2). Schackert et al. also described RPA class 1 as a favorable factor for prolonged postoperative survival, along with other factors such as a small number of metastases and adjuvant Rtx [25]. As a consequence, the RPA score can be a useful tool when considering indications for surgery.

#### Preoperative vs. postoperative deficit

Only 8.0% in the motor eloquent and 10.3% in the non-motor-eloquent group with preoperative deficits suffered permanent deficit (Figure 6B). The neurological status worsened after surgery in only 17.0% of all patients. When Walter et al. treated metastases in the central region in 20 patients, incidence of postoperative paresis was 15.0% [26]. Paek et al. investigated 208 patients with motor eloquently located metastases, and only 8.0% worsened postoperatively in their KPS but 6 to 19% showed a new surgery-related permanent neurological deficit [14]. Note that even new minor motor deficits can be assigned to the same KPS as preoperatively. In motor eloquently located gliomas, several studies showed similar results [27-29].

#### Different treatment strategies vs. new postoperative deficit

Figure 7 emphasizes the significant role of previous Rtx leading to significantly higher risk for new postoperative motor deficits in patients with motor eloquently located brain metastases (p = 0.0116). By contrast, Ctx seems to have no impact on this.

In the motor eloquent group and non-motor-eloquent group we found a significant relation between performed preoperative treatment strategies (p = 0.0122 vs. 0.045). Despite the small number of patients, we can claim that Rtx, alone or in combination with Ctx, increases the risk of new postoperative deficits, especially in motor eloquent metastases (Figure 7). Nevertheless, surgery can be a useful tool, especially in cases of symptomatic mass effect [30,31]. In these cases, IOM can minimize risk of postoperative deficits [3].

### Limitations

The number of enrolled patients is still small in some subgroups, and in some cases — for instance, the relation between RPA class and survival — may have cost the study statistical significance. The retrospective analysis of our data is another major limitation.

## Conclusions

Surgical resection of brain metastases is a safe procedure despite still harboring considerable risks for the patients. But some factors influence the postoperative outcome more than expected. Preoperative Rtx, motor eloquent tumor location, and RPA class play a significant role in the likelihood of new postoperative motor deficits. Thus, on the basis of this study, indication for surgical resection has to be considered carefully in these subgroups and discussed carefully in interdisciplinary oncological conferences.

## Abbreviations

ANOVA: Nonparametric one-way analysis of variance; CST: Corticospinal tract; Ctx: Chemotherapy; GTR: Gross total resection; IOM: Intraoperative neuromonitoring; KPS: Karnofsky performance scale; MEP: Motor evoked potentials; MRI: Magnetic resonance imaging; RPA: Recursive partitioning analysis; Rtx: Radiotherapy; SD: Standard deviation; STR: Subtotal resection; TIVA: Total intravenous anesthesia; UR: Unexpected residual; WBRT: Whole brain radiation therapy.

## Competing interests

The authors declare that they have no competing interest that affects this study. The study was completely financed by institutional grants from the Department of Neurosurgery. The authors report no conflict of interest concerning the materials or methods used in this study or the findings specified in this paper.

## Authors’ contributions

TO, MS, and JG were responsible for data acquisition, performed data analysis and clinical assessment. TO performed statistical analyses, drafted and approved the manuscript. MS and JG approved and corrected the final version of the manuscript. BM is responsible for the original idea, supervised the study, but also approved and corrected the final version. FR supervised the study, revised the manuscript, approved and corrected the final version. SK was responsible for the original idea, the concept and design. SK was responsible for data acquisition, handled the acquired data, and performed literature research as well as statistical analyses. SK drafted the manuscript and approved its final revision. All authors read and approved the final manuscript.

## Authors’ information

All authors are strongly involved in the treatment of brain tumors including awake surgery, preoperative mapping, and intraoperative neuromonitoring in a specialized neurooncological center. BM is chairman and FR is vice chairman of the department.

## Pre-publication history

The pre-publication history for this paper can be accessed here:

http://www.biomedcentral.com/1471-2407/14/21/prepub
